# Cancer risks in childhood and adolescence among the offspring of immigrants to Sweden

**DOI:** 10.1038/sj.bjc.6600227

**Published:** 2002-05-06

**Authors:** K Hemminki, X Li

**Affiliations:** Department of Biosciences at Novum, Karolinska Institute, 141 57 Huddinge, Sweden

**Keywords:** brain tumour, leukaemia, lymphoma, non-Hodgkin's lymphoma, infection

## Abstract

We used the nation-wide Swedish Family-Cancer Database to analyse the risk of nervous system tumours, leukaemia and non-Hodgkin's lymphoma in age groups 0–4 and 0–19 years among Swedish-born offspring of immigrants. The study included 850 000 individuals with an immigrant background, including European, Asian and American parents. We calculated standardised incidence ratios for the above three malignancies using Swedish offspring as a reference. Subjects were grouped by region or by selected countries of parental origin. No group differed significantly from Swedes in the occurrence of nervous system neoplasm or leukaemia. Offspring of Yugoslav fathers (SIR 2.27) and Turkish parents were at increased risk of non-Hodgkin's lymphoma. The highest risk was noted for non-Hodgkin's lymphoma among young offspring (0–4 years) of two Turkish parents (6.87). The currently available limited data on rates for childhood non-Hodgkin's lymphoma in these countries do not explain the risk in the offspring of immigrants. Yugoslavs and Turks are recent immigrant groups to Sweden, and their offspring have been subject to much population mixing, perhaps leading to recurring infections and immunological stimulation, which may contribute to their excess of lymphomas.

*British Journal of Cancer* (2002) **86**, 1414–1418. DOI: 10.1038/sj/bjc/6600227 www.bjcancer.com

© 2002 Cancer Research UK

## 

Nervous system tumours, leukaemia and lymphoma are the most common neoplasia in childhood and adolesence before age 20 years (Draper *et al*, 1994; Gurney *et al*, 1996; Linet *et al*, 1999; Little, 1999; Stiller and Parkin, 1990, 1996). The only risk factor with a large population attributable impact is Epstein-Barr virus (EBV) in relation to proportion of Burkitt's lymphomas in endemic areas of Africa, and some 30% of childhood non-Hodgkin's lymphomas and Hodgkin's disease in North America and Europe (IARC, 1997b; Pisani *et al*, 1997; Jaffe *et al*, 2001). Family history is a risk factor for nervous system cancer and lymphoma but it affects a small proportion of all cases (Hemminki *et al*, 2000; Hemminki and Mutanen, 2001). Migrant studies have indicated the importance of environment in cancer aetiology (Parkin and Khlat, 1996) with, however, almost exclusive focus on adult cancers of, primarily, the first generation immigrants (Haenszel and Kurihara, 1968; McMichael *et al*, 1980; Steinitz *et al*, 1989; Parkin *et al*, 1990; Balzi *et al*, 1993; Iscovich and Parkin, 1997; Parkin *et al*, 1997; McCredie *et al*, 1999a,b). Exceptions include childhood cancer studies among the offspring of south Asian immigrants to England (Varghese *et al*, 1996; Cummins *et al*, 2001).

Some 10% of the Swedish population of nine million are foreign-born and they have close to one million offspring born in Sweden. The largest groups of immigrants have come from the Nordic Countries, particularly, Finland, Eastern and Western Europe, and from Asia (Table 1

**Table 1:**
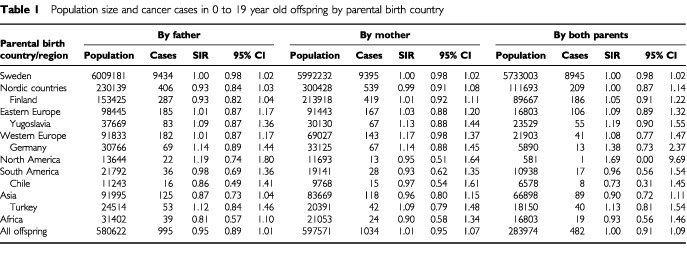
Population size and cancer cases in 0 to 19 year old offspring by parental birth country

). The most recent immigrant groups are the Chileans and Yugoslavs who came in large numbers in the 1970s, and more recently Turks, Kurds, Iranians and other groups from the Near East, escaping persecution, poverty and wars. We have used the nationwide Swedish Family-Cancer Database, in which the birth country of each individual is recorded, to study the incidence up to age 20 years among the Sweden-born offspring of immigrants of the three common malignancies, nervous system tumours, leukaemia and non-Hodgkin's lymphoma. Hodgkin's disease was initially included but because of the small number of cases and the absence of significant associations, the results are not presented.

## SUBJECTS AND METHODS

The Swedish Family-Cancer Database was initially created in the middle of the 1990s by linking an administrative family register on all Swedish families to the Swedish Cancer Registry (Hemminki, 2001; Hemminki *et al*, 2001). For each child there are data on both parents at the time of birth. Each person has been assigned a unique technical identification number (which is different from the national identification number, ‘personal number’), allowing construction of families for example through the mother. The Database includes all persons born in Sweden after 1931 with their biological parents, totalling over 10.2 million individuals. It has been updated at the beginning of 2001 to include cancers from the nationwide Swedish Cancer Registry from the years 1958 to 1998. The Database is organized in 3.2 million families, with parents and offspring. Among the offspring, 580 904 have a mother, 597 571 have a father and 857 051 have any foreign-born parent.

The completeness of cancer registration in the 1970s has been estimated to be over 95%, and is now considered to be close to 100%. The Swedish Cancer Registry is based on compulsory notification of cases (Centre for Epidemiology, 2000). In the Registry, the diagnostic data are coded according to the 7th revision of the International Classification of Diseases (ICD-7). Leukaemia included lymphoid, myeloid, monocytic and other leukaemias.

The parental information was classified according to the country of birth, and the incidence of cancer was calculated for their 0 to 4 or 0 to 19 year old offspring. ‘Eastern Europe’ included the Czech Republic and all European countries east of it, but Greece was included in ‘Western Europe’. All incidence rates were based on the data in the Family-Cancer Database. Follow-up was started at birth or 1 January, 1961, whichever came latest. Follow-up was terminated at age 5 or 20, diagnosis of cancer, emigration, or the closing data of the study, 31 December, 1998. Standardised incidence ratios (SIRs) were calculated as the ratio of observed (O) to expected (E) number of cases. The expected numbers were calculated from 5-year-age-, sex-, 10 year-period-, region- (three largest cities, south Sweden and the rest) and tumour type-specific standard incidence rates. The incidence among all Swedish offspring was used as the reference rate. Confidence intervals (95%CI) were calculated assuming a Poisson distribution.

## RESULTS

The present study covered years 1961 to 1998 from The Family-Cancer Database. The offspring of the Swedish natives constituted by far the largest group of offspring in the study, about six million individuals and close to 160 million person-years (Table 1). Close to 0.6 million offspring had a foreign-born father or mother; combined there were 0.86 million offspring with at least one parent born abroad. Offspring of Finnish parents were by far the largest group, followed by those of Yugoslav and German parents. The number of offspring was about equal among immigrants from Eastern and Western Europe and from Asia. However, Asians have come recently and the mean follow-up time for their offspring was relatively short. The total number of cancers in age group 0 to 19 years was over 10 000, but only about 2500 cancers came from families of a foreign father/mother. The overall cancer incidence was not increased for offspring of immigrants. There was no significant decrease in risk. Below, we show the results on the three most common neoplasms, with the parental country or region of origin. The five specific countries were selected as origins of the largest immigrant groups from the particular regions.

Table 2

**Table 2:**
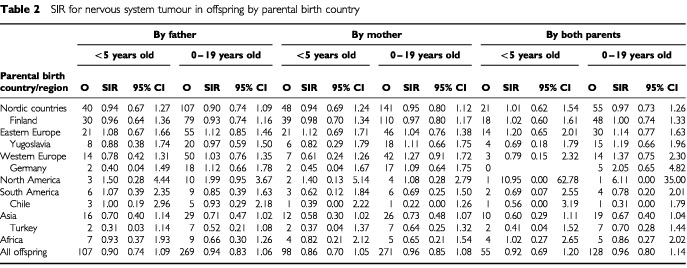
SIR for nervous system tumour in offspring by parental birth country

shows SIRs for offspring nervous system cancer by parental country of origin. None of the SIRs deviated significantly from unity. Risks for leukaemia are shown in Table 3

**Table 3:**
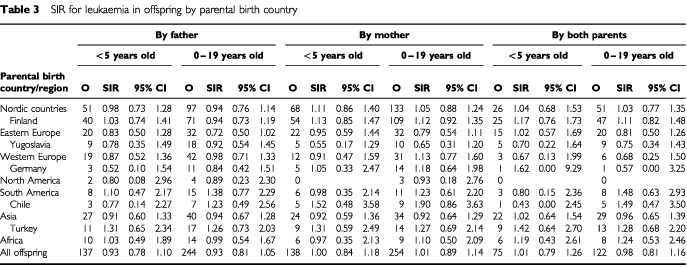
SIR for leukaemia in offspring by parental birth country

but no significant differences were observed.

The risk of non-Hodgkin's lymphoma was increased in 0 to 19 year old offspring of Yugoslav fathers (SIR 2.27), and among all 0 to 4 year old offspring of Asian parents (Table 4

**Table 4:**
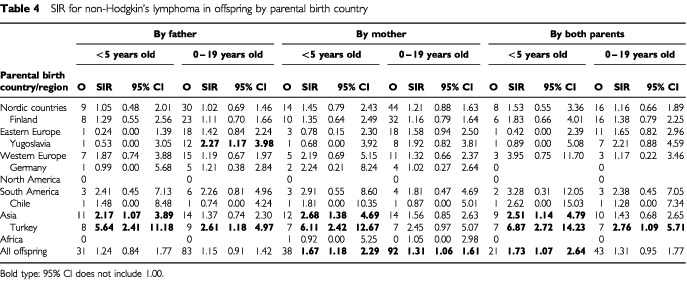
SIR for non-Hodgkin's lymphoma in offspring by parental birth country

). The high SIRs for the latter group were largely explained by Turkish immigrant families. When both parents were Turkish the SIRs were 6.87 among the 0 to 4 year old and 2.76 among the 0 to 19 year old. No sibling pairs with non-Hodgkin's lymphoma were found in Yugoslav or Turkish families. The high SIRs in Turkish families caused an increase for all offspring of immigrants.

## DISCUSSION

Most migrant studies on adult cancers have shown that the cancer incidence in migrants moves towards the level of the host population in one or two generations, which has been interpreted as support for the environmental aetiology of cancer (IARC, 1990; Parkin and Khlat, 1996; McCredie, 1998). The data on adult immigrants to Sweden are consistent with such data (Hemminki and Li, 2002; Hemminki *et al*, 2002). The data on childhood cancer are limited but those suggest for the offspring of south Asian immigrants in the UK suggest that differences from the native population exist for some neoplasms (Varghese *et al*, 1996; Cummins *et al*, 2001). Similarly, the relatively young second-generation Israelis seem to maintain some of the cancer rates from the countries of their parental origin (Iscovich and Parkin, 1997; Parkin *et al*, 1997). Inherited susceptibility or slow integration process of immigrants may explain the risks in the second generation.

The geographic variation in the incidence of nervous system tumours, of which most are brain tumours, is relatively small, at about 2–3-fold (Stiller and Parkin, 1996; Little, 1999). The Nordic Countries, including Sweden, have the highest annual rates of over 30 per million, compared to those in other European countries and North America (20–30 per million) and in Africa and Asia (10–20 per million). Our results showed no differences for any groups of offspring from immigrant families, and all the groups with reasonable numbers of cases showed SIRs very close to unity. There is evidence that at least part of the past increase in the incidence of childhood brain tumours in Western countries can be ascribed to changes in diagnostic and reporting practice (Hemminki *et al*, 1999; Linet *et al*, 1999), which may suggest, together with the observed similar rates between various ethnic groups in Sweden, that the real international difference in the rates of childhood brain cancer may be less than the cited 2–3-fold.

The international rates for childhood leukaemia also vary to only a limited extent, some five-fold (Stiller and Parkin, 1996; Little, 1999). The highest rates are recorded for Costa Ricans and Los Angeles Hispanics (over 50 per million per year), with intermediate rates for North American whites, Europeans and South Americans (30–50 per million), and the lowest rates for Indians, Arabs and Africans (10–30 per million). In Europe, the rates differ irregularly, from the highest in the UK, some parts of Italy and Switzerland through Sweden, Denmark, the Netherlands, France, Czech and Poland to the low rates in Norway, Finland, some parts of Italy and the Baltic Countries (IARC, 1997a). Among the 63 non-overlapping European cancer registries, with data on 0 to 19 year old acute lymphoid leukaemia, listed in the Cancer Incidence in Five Continents (CI5VII, Electronic Database of Cancer, IARC 1997), Sweden ranked 19th among males and 16th among females. In the present study, we found that the offspring of immigrants did not differ from Swedes in their leukaemia rates, suggesting that the international variation in childhood leukaemia rates, if real, is probably mainly due to environmental causes.

In contrast to the above two malignancies, non-Hodgkin's lymphoma shows a large international variation in incidence. However, this is mainly due to Burkitt's lymphoma, and if it is excluded, the variation is about five-fold (Stiller and Parkin, 1990). The diagnostic criteria for lymphomas have changed extensively over the years and, undoubtedly, there are international differences, e.g., between non-Hodgkin's lymphoma and Hodgkin's disease (Jaffe *et al*, 2001). Re-examination of the pathological specimens have lead to the adjustment of the original Hodgkin's disease diagnosis to non-Hodgkin's lymphoma in some 10% or more of the cases (Travis *et al*, 1991; Martinsson *et al*, 1992; Abrahamsen *et al*, 1997; Kumar *et al*, 1997; Jaffe *et al*, 2001). Thus, the international rates for lymphomas should be viewed with some reservation. We have analysed also childhood Hodgkin's disease among immigrant offspring, but none of the present findings can be explained by the possible misclassification of Hodgkin's disease and non-Hodgkin's lymphoma. An interesting aspect of childhood non-Hodgkin's lymphoma is that the incidence rates have remained stable in Europe and the USA even though the adult disease has been the most rapidly increasing neoplasia (Linet *et al*, 1999; Parkin *et al*, 1999; Weidmann *et al*, 1999; Howe *et al*, 2001). It may be noted that Sweden is a high-risk country for adult non-Hodgkin's lymphoma but at an intermediate-risk for the childhood disease (IARC, 1997a). Sweden ranked 29th among male and female non-Hodgkin's lymphoma rates at ages 0 to 19 years among 63 European cancer registries (IARC 1997a; Electronic Database).

Our data showed that offspring of Yugoslav fathers were at increased risk of non-Hodgkin's lymphoma. The rates for non-Hodgkin's lymphoma among 0 to 19 year old people were similar in Yugoslavia and Sweden (IARC 1997a). In the present study, the highest risks were observed for children (particularly 0 to 4 year old) of Turkish parents, the SIR being as high as 6.87 when both parents were Turkish, as they were the majority. No incidence data are available on non-Hodgkin's lymphoma from Turkey but judging from the incidence reported for Iraq and Israel (Stiller and Parkin 1990), the indigenous rates in Turkey may exceed Swedish rates somewhat, but not enough to explain the high rates observed in immigrant families. According to GLOBOCAN 2000, the incidence of childhood non-Hodgkin's lymphoma is lower in Turkey than in Sweden, or at the same level, but for Turkey data were extrapolated from the regional Turkish incidence rates (IARC, 2001). The incidence of non-Hodgkin's lymphoma in adult immigrants from Yugoslavia and Turkey to Sweden does not differ from the Swedish rates (Hemminki *et al*, 2002). The present data are consistent with an excess of non-Hodgkin's lymphoma in offspring of South Asians in England and of Asian and African Jews in Israel (Varghese *et al*, 1996; Iscovich and Parkin, 1997; Cummins *et al*, 2001).

The present data on non-Hodgkin's lymphoma suggest that the offspring of immigrant groups, most clearly those of Yugoslavs and Turks, develop an increased incidence in Sweden rather than inherit it from their parents. An immigrant mother did not appear to be a more important risk factor than an immigrant father, making it perhaps less likely that the foetus–mother immunological adjustment had been impaired or that infections during pregnancy played a role. Yet the conclusion needs to be guarded because for many affected offspring both parents were immigrants, particularly among Turks. There were common features in these imigrant groups; they had arrived relatively recently, and Turks with the highest risk were the latest arrivals; culturally, they are more distant from the Swedes than the previous immigrant groups, and the common compatriot marriages indicated that they immigrated as couples or sought a spouse among their own ethnic group. These factors suggest that these immigrant families were between a social and cultural transition, exposing offspring to contacts with different ethnic groups. This may involve a large amount of population mixing, infections and antigenic stimulation, which are suggested risk factors for childhood leukaemia and lymphoma (Kinlen, 1995; Kinlen *et al*, 1995; Greaves, 1999; Little, 1999; Kinlen and Bramald, 2001).

In summary, most offspring of immigrants to Sweden shared the childhood cancer experience of the Swedes. Offspring of Yugoslav and Turkish parents had an increased risk of non-Hodgkin's lymphoma, perhaps because of their immune response to new infections.
